# Effects of Cu^2+^/Zn^2+^ on the electrochemical performance of polyacrylamide hydrogels as advanced flexible electrode materials†

**DOI:** 10.1039/d2ra02391a

**Published:** 2022-06-30

**Authors:** Syed Faizan, Luqman Ali Shah, Bakhtawara, Daixin Ye, Fawad Ahmad, Musammir Khan, Muhammad Ismail

**Affiliations:** Polymer Laboratory, National Centre of Excellence in Physical Chemistry, University of Peshawar Peshawar 25120 Pakistan luqman_alisha@uop.edu.pk luqman_alisha@yahoo.com +92919216671 +92919216766; Institute for Sustainable Energy, College of Sciences, Shanghai University Shanghai 200444 PR China; Department of Chemistry, University of Wah Quaid Avenue, Wah Cantt. Rawalpindi Punjab Pakistan; Department of Chemistry, Women University Swabi Khyber Pakhtunkhwa Pakistan

## Abstract

Previously, solid-state electrode materials have been utilized for the fabrication of energy storage devices; however, their application is impeded by their brittle nature and ion mobility problems. To address issues faced in such a modern era where energy saving and utility is of prior importance, a novel approach has been applied for the preparation of electrode materials based on polyacrylamide hydrogels embedded with reduced graphene oxide and transition metals, namely, Cu^2+^ and Zn^2+^. The fabricated hydrogel exhibits high electrical properties and flexibility that make it a favorable candidate to be used in energy storage devices, where both elastic and electrical properties are desired. For the first time, a multi-cross-linked polyacrylamide hydrogel was constructed and compared in the presence of other electro-active materials such as reduced graphene oxide and transition metals. Polyacrylamide hydrogels embedded with reduced graphene oxide demonstrate excellent electrical properties such as specific capacitance, least impedance, low phase angle shift and AC conductivity of 22.92 F g^−1^, 2115 Ω, 2.88° and 0.67 μδ m^−1^ respectively as compared to Cu^2+^- and Zn^2+^-loaded hydrogels, which block all available active sites causing an increase in impedance with a parallel decrease in capacitance. The capacitance retention and coulombic efficiency calculated were 88.22% and 77.23% respectively, indicating high stability up to 150 cycles at 0.1 A g^−1^. Storage moduli obtained were 10.52 kPa, which infers the more elastic nature of the hydrogel loaded with graphene oxide than that of other synthesized hydrogels.

## Abstract 2

A novel approach has been applied for the preparation of electrode materials based on polyacrylamide hydrogels embedded with reduced graphene oxide and transition metals, Cu^2+^ and Zn^2+^. The fabricated hydrogel exhibits high electrical properties and flexibility.

## Graphical abstract

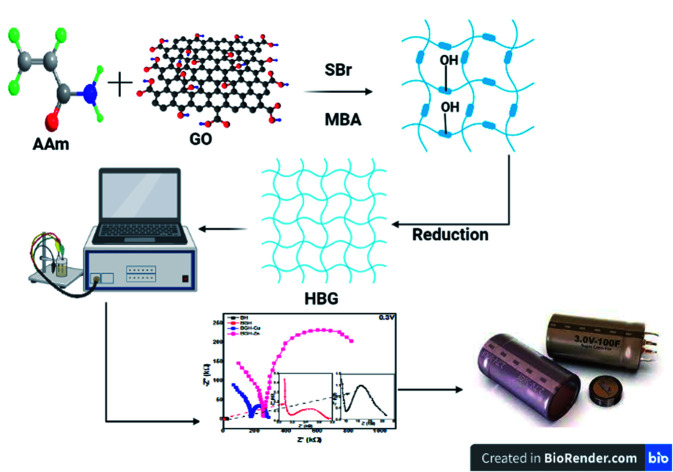

## 1. Introduction

To facilitate human life on earth, various electrical and electronic devices such as home appliances, mobile phones, and laptops have been invented by researchers. To impart electrical and electrochemical properties to such devices, different materials such as activated carbons and conducting polymers such as polyaniline^1^ and polypyrrole^2^ for energy storage, saving and its utility have been used. However, nowadays, researchers are motivated to develop devices such as robots, wearable devices, solar cells and portable electronic devices, which was not satisfied from the use of conducting polymers and carbon-based materials due to high prices, synthesis problem, low stability, and ion mobility issues. Moreover, when these polymers are used in electrode applications, they will be deposited in the form of films, which often become brittle with the passage of time.^3^ Therefore, the demand for having flexible nature and maximum ion mobility is increasing with the advancement, which cannot be induced by using it in the composite form.

To overcome the ion mobility and flexibility problem duly faced by electrode materials, the researchers focused on the synthesis of electrochemical active hydrogels.^4^ Hydrogels are 3D network structures of polymer chains cross linked by physical or chemical linking. They can absorb an excessive amount of water without causing cracking in the skeleton network. The intelligent behavior makes these hydrogels to be used in biosensing,^5^ self-healing ability,^6,7^ radiotherapy,^8^ drug delivery,^9^ water treatment,^10–12^ electrochemical stain removal,^13^ and supercapacitance.^14,15^ Other specific properties such as conductivity, flexibility, consecutive bending and twisting make them promising materials to be used in flexible electrodes for energy storage devices and wearable electronics.^16^ Hydrogels have been applied for the synthesis of advanced electrode materials covering nearly all the drawbacks such as ion mobility, flexibility, stretching and high cost.

Many advances have been made in the field of portable and wearable devices, but these devices can be damaged or broken down when folded or bent while using. Therefore, its flexibility is desired in such devices that can tolerate the stress applied and is not broken down. Hydrogels contain an excessive amount of water within their 3D polymer matrix, causing resistance to fracture when certain stress has been applied. Hydrogels have been used as electrolytes as well as electrode materials. Polyvinyl alcohol is generally used as an electrolyte, but its application in wearable electronics is negotiated because of its low mechanical strength. For hydrogel synthesis, cross-linking (physical or chemical) plays an important role in the 3D matrix. In previous research, polyacrylamide-based hydrogels are cross-linked *via* a chemical cross-linker *i.e. N*,*N*′-methylenebisacrylamide (MBA).^17^ However, the corresponding polyacrylamide hydrogel was poor in its electrical conductivity and mechanical strength, as there is no active material present for the transportation or storage of charges.

Therefore, both mechanical strength and electrochemical/electrical properties are of prime importance. The mechanical strength can be controlled by multi-crosslinking in the polymer matrix. Like flexibility can be imparted by the use of physical cross-linkers, however mechanical properties can be imparted by the use of chemical cross-linkers or sodium montmorillonite clay.^18^ In the same perspective to enhance the electrical conductivity, sodium borate anhydrous (SBr) was used as a physical cross-linker, which utilizes sodium ions responsible for the conduction of current.^19^

Among certain electrode materials, graphene-based materials are of prime importance due to their superior chemical stability, electrical conductivity, substantial surface area, and high mechanical strength.^20^ Arun. K. Nandi *et al.* synthesized a poly[3-(2-hydroxyethyl)-2,5-thienylene]-*grafted* reduced graphene oxide and polyaniline (PANI) rectangular nano-pipe in the presence of amino-functionalized reduced graphene oxide for dye-sensitized solar cells.^21,22^ Previously, researchers have focused on the synthesis of graphene sheets to use them in electrochemical applications; besides, intrinsic properties such as binding and mechanical strength of graphene-based materials, self-contained hydrophilic groups and conducting frameworks made them promising electrode materials. The problem with graphene-based materials is their out-of-plane pores, which hinder ion mobility through the graphene sheet. Therefore, in order to cover up the problem of ion mobility and electrode kinetics, in-plane pores are introduced into graphene hydrogels; as a result, the electrolyte ion is capable of directly moving into graphene hydrogels enhancing charge transfer and decrease the path length for ion transport.^23,24^ Therefore, the porosity, both in-plane and out-of-plane, will enhance the electrode kinetics.^20^

Different metal oxides such as Ni(OH)_2_, NiO, and Co_3_O_4_ were used for producing electrodes having high theoretical capacitance and cost-effectiveness.^25–27^ However, due to their low conductivity, fast electron transport is not possible, and therefore, its conductivity can be improved by adding a variety of carbon materials such as conducting polymers,^2^ active carbons,^28^ carbon nanotubes,^29^ and graphenes.^30^ The advantages of porous graphene-based hydrogels such as enhanced transport property and mechanical flexibility with various metal hydroxides make them promising candidates used for advanced electrode materials.

In this work, we fabricated pure (HB), graphene oxide-doped (HBG), Cu^2+^-doped (HBG-Cu), and Zn^2+^-doped (HBG-Zn) multi-cross-linked polyacrylamide hydrogels having high electrical conductivity and good flexibility. Graphene oxide-doped hydrogels were reduced before the electrochemical investigations. The reduction of graphene oxide was carried out due to the less C/O on the surface of GO, which acts as a barrier for the transportation of charges.^31^ Polyacrylamide hydrogels embedded with reduced graphene oxide demonstrate excellent electrical properties such as specific capacitance, least impedance, and low phase angle shift as compared to Cu^2+^- and Zn^2+^-loaded hydrogels, which block all available active sites causing an increase in impedance with a parallel decrease in capacitance. The fabricated hydrogels exhibit high electrical and mechanical properties with flexible nature, which make them suitable candidates to be applied on an industrial scale in flexible electrode materials.

## 2. Experimental

### 2.1 Applied materials

Acrylamide (AAm), *N*,*N*′-methylenebisacrylamide (MBA, 97%), and ascorbic acid were purchased from BDH, England. Sodium borate anhydrous was obtained from Daejung Chemicals, and ammonium persulphate (APS, 99%), tetramethylethylenediamine (TEMED), potassium dichromate (KMnO_4_), hydrogen peroxide (H_2_O_2_), nitric acid (HNO_3_), sulphuric acid (H_2_SO_4_), phosphoric acid (H_3_PO_4_), and hydrochloric acid (HCl) were received from Sigma Aldrich Belgium. Graphite powder, copper nitrate, and zinc nitrate were obtained from Scharlau Chemicals. The solvent used throughout the experimental process was Milli-Q distilled water, ELIX UV 5.

### 2.2 Synthesis of graphene oxide

Graphene oxide (GO) was synthesized by modified Hummer's method using a graphite powder.^32^ A 50 mL mixture of 70%, 20%, and 10% of H_2_SO_4_, H_3_PO_4_, and HNO_3_ respectively was taken in a beaker and charged with 1 g of graphite powder under stirring. After a homogenous dispersion of graphite flakes was obtained, 6 g of KMnO_4_ (oxidizing agent) was added keeping temperature less than 5 °C. After that, the beaker was placed in an oil bath at a temperature of 45 °C for 2 hours. To prevent a rapid rise in temperature, the beaker was placed in an ice bath while pouring 100 mL of deionized water and the reaction temperature was maintained at 85 °C for 1 hour. The whole experiment was performed under continuous stirring. To bring the reaction to an end, 120 mL double-deionized water and 15 mL of 30% H_2_O_2_ solution were added to the reaction mixture. The reduction of manganese dioxide and permanganate was observed by change in the color to light yellow. The solution temperature was decreased to room temperature and 25 mL of 10% HCl solution was added to the solution to remove metal ions. The mixture was centrifuged several times and washed with Milli-Q distilled water in order to increase the acidic pH to pH 7.0. Once the solution reached a pH value of 7, the solution was poured into a Petri dish, and placed in an oven at 85 °C for 24 hours. After incubation, thin films of GO were synthesized and stored for further use.

### 2.3 Fabrication of multi-cross-linked polyacrylamide hydrogels

The fabrication of multi-cross-linked polyacrylamide hydrogels was carried out by free radical polymerization. A stock solution was prepared by dissolving 6 g of acrylamide monomer in 40 mL of Milli-Q distilled water. Subsequently, 80 mg each of MBA, sodium borate anhydrous, and montmorillonite clay were added to the stock solution and sonicated until a homogeneous solution was obtained. The stock solution was equally distributed into four beakers marked as a, b, c, and d. Then, 20 mg of GO was charged to all beakers except beaker a, and further 20 mg of copper nitrate and 20 mg of zinc nitrate were added to beakers c and d respectively. Finally, polymerization was initiated by adding 20 μL TEMED followed by 2 mL of 4% APS solution to each beaker. The hydrogels were synthesized within three minutes at room temperature. The synthesized hydrogels were reduced using 20 mg mL^−1^ solution of ascorbic acid at 40 °C for 24 hours. The successful reduction was noted by color change from transparent to yellow and brown to black for sodium borate and GO-based hydrogels respectively. Fabricated hydrogels a, b c, and d were marked as HB, HBG, HBG-Cu, and HBG-Zn respectively.

### 2.4 Preparation of working electrodes

A thin copper wire was taken and immersed in synthesized polyacrylamide hydrogels in the swollen state. The hydrogel along with a copper wire was placed in an oven at 40 °C for 12 hours to remove free water and used as a working electrode.

### 2.5 Electrochemical test of hydrogel electrodes

The prepared polyacrylamide-based hydrogels were used as working electrodes, gold as the counter electrode, and Ag/AgCl as the reference electrode in a three-electrode system. The electrolyte used throughout the electrochemical study was 0.5 M H_2_SO_4_. The electrochemical properties of hydrogels were compared and the hydrogel with higher conductivity, capacitance, and low resistance was preferred for flexible electrode applications.

### 2.6 Characterization

The crystalline structure of graphene oxide was determined by X-ray diffraction using JDX-3532, JEOL, Japan. Functional groups of the synthesized hydrogels were determined by FT-IR spectroscopy using Shimadzu, IR Presigue-21 in the range of 400–4000 cm^−1^. The surface morphology of hydrogels was determined by scanning electron microscopy (SEM JEOL JSM-5910S), and elemental diffraction analysis was performed using a SEM JSM-IT-100. The samples for analysis were dried at 50 °C in a vacuum oven and further ground a powder form after synthesis. The electrochemical properties of the synthesized hydrogels were investigated using a Metro Ohm Auto lab PGSTAT 302. The AC conductivity of the synthesized hydrogel was determined using a 2401 KEITHLEY source meter. However, the mechanical property was elaborated using an Anton Paar MCR 301 Rheometer.

## 3. Results and discussion

### 3.1 Characterization of the as-synthesized polyacrylamide hydrogels

The crystalline structure of prepared GO (used as a cross-linker) was characterized by XRD analysis (Fig. S1†). A sharp diffraction peak at 2*θ* Braggs' angle around 10.11° corresponds to the hexagonal crystalline structure attributed to the (001) facet with a d-spacing of 8.75 Å.

**Fig. 1:**
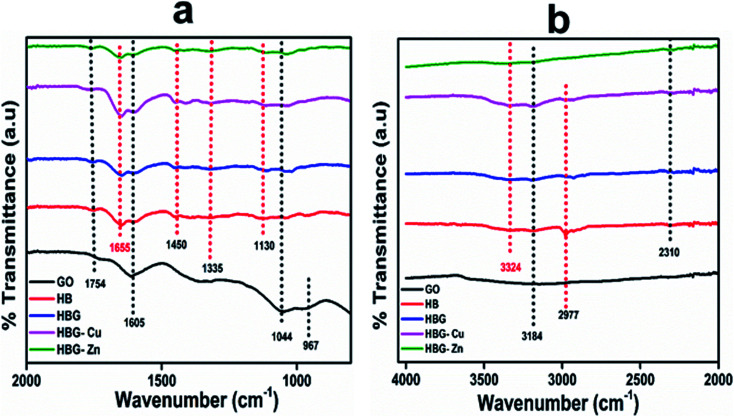
FTIR spectra of prepared polyacrylamide hydrogels from (a) 800–2000 cm^−1^ and (b) 2000–4050 cm^−1^.

For the confirmation of polymerization and hydrogel formation, the FTIR spectra were recorded, as shown in Fig. 1. All the peaks associated with multi-cross-linked polyacrylamide hydrogels were present, which clearly indicate the successful fabrication of polymer hydrogels. The peaks at 3184 cm^−1^, 2977 cm^−1^, 1754 cm^−1^, 1605 cm^−1^, and 1044 cm^−1^ correspond to –OH, –CH, C=O, C=C, and C–O–C stretching vibration modes, while the peaks at 1335 cm^−1^ and 967 cm^−1^ correspond to –OH and C=C bending vibrations of graphene oxide respectively. For all multi-cross-linked polyacrylamide hydrogels, a broad doublet peak appeared at 3324 cm^−1^ and 3184 cm^−1^ due to the stretching vibration of –OH and –NH groups. The peak shifting for particular functional groups in the range of 2400 to 3600 cm^−1^ (inset) for HBG-Cu and HBG-Zn indicates the existence of possible inter actions between entrapped metals and polymer chains. All the fabricated hydrogels exhibited a peak at 2310 cm^−1^ due to CO_2_ formed after reduction of C–O groups by ascorbic acid.^33,34^

**Fig. 2:**
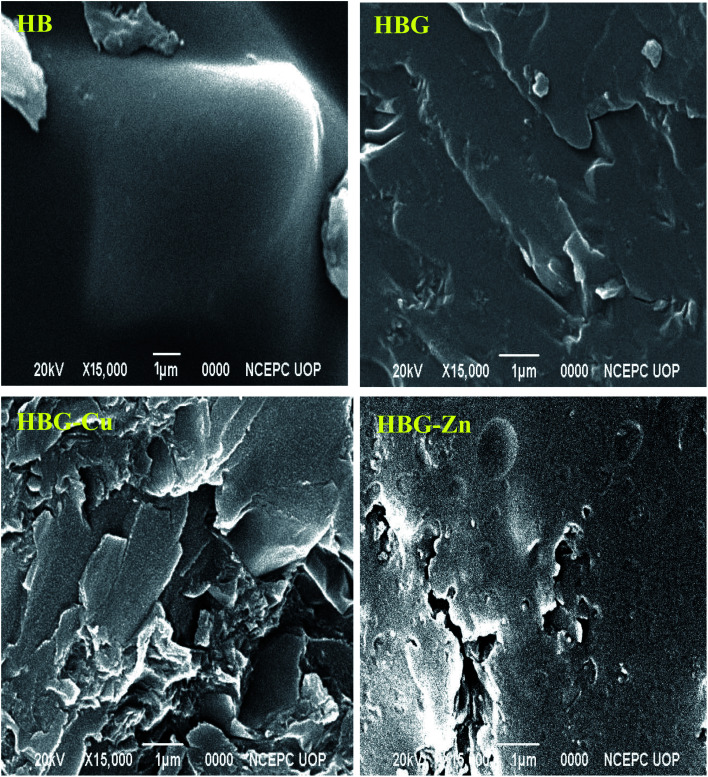
Scanning electron microscopic images of the prepared multi-cross-linked polyacrylamide hydrogels.

The surface morphology of multi-cross-linked hydrogels was elaborated by SEM analysis (Fig. 2). A tight, wavy, and dense surface was obtained for HB, while after addition of GO followed by reduction, the structure became wrinkled, rough, and have lamellar morphology for HBG. The change in surface morphology clearly indicates the involvement of extra interactions in hydrogels due to rGO. The incorporation of Cu^2+^ and Zn^2+^ changes the hydrogel surface from smooth to rough having cracks. This appearance of cracks is due to the interaction of Cu^2+^ (in HBG-Cu) and Zn^2+^ (in HBG-Zn) with GO and hydrogel networks. Fig. S2† shows the smooth surface for HB and HBG, whereas for metal-loaded gels, the surface seemed to be rough under visible light.

The EDX analysis was performed to determine the elemental composition of prepared HB, HBG, HBG-Cu, and HBG-Zn hydrogels, as shown in Fig. S3,† and the atomic percentage of each atom is tabulated in Table S1.† It has been inferred that carbon, nitrogen, and oxygen peaks were observed in the EDX spectra for all polyacrylamide hydrogels due to the presence of acrylamide monomers. However, HBG-Cu and HBG-Zn give an additional peak of Cu and Zn respectively, which clearly elaborates the specific transition metal loading in the hydrogel 3D structure.

### 3.2 Proposed mechanism

**Scheme 1:**
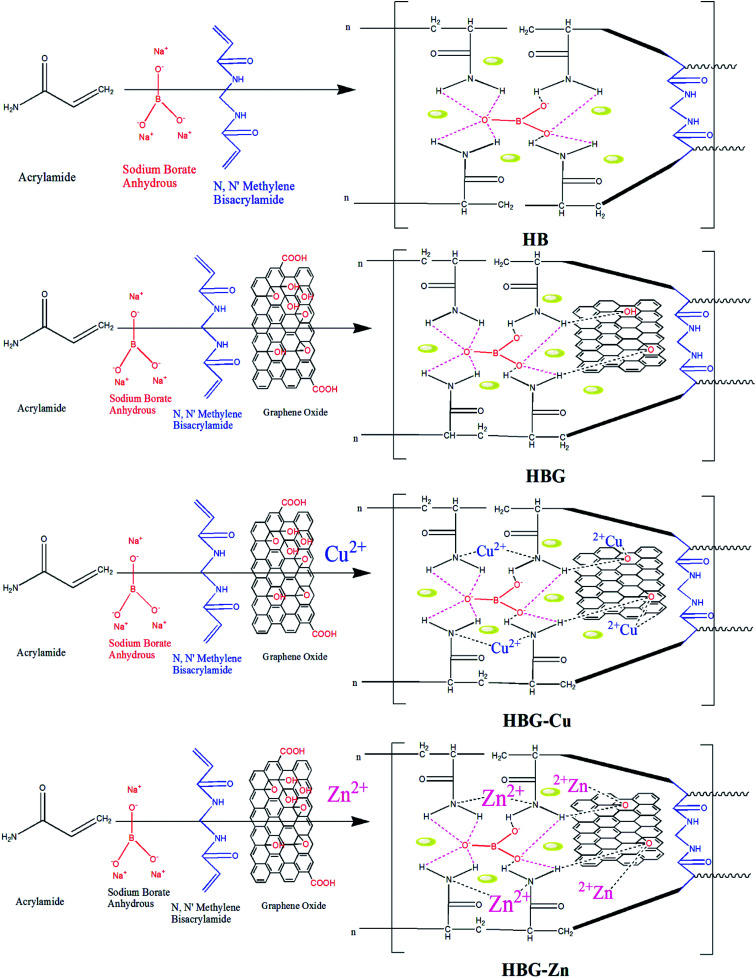
Possible inter-network interactions in multi-cross-linked polyacrylamide hydrogels.

The proposed mechanism with all possible interactions in hydrogels is shown in Scheme 1. The growing polyacrylamide hydrogel comprises MBA, SBr, and reduced graphene oxide (rGO), where MBA act as a chemical cross linker and combines the polymer chains *via* chemical bonds, whereas SBr and rGO develop electrostatic interactions and hydrogen bonding respectively in polymer networks. Furthermore, the addition of Cu^2+^ and Zn^2+^ to hydrogels further enhances the inter network interactions and confirms the existence of these transition metal ions in the hydrogel 3D structure.

### 3.3 Electrochemical analysis

#### 3.3.1 Cyclic voltammetry

**Fig. 3:**
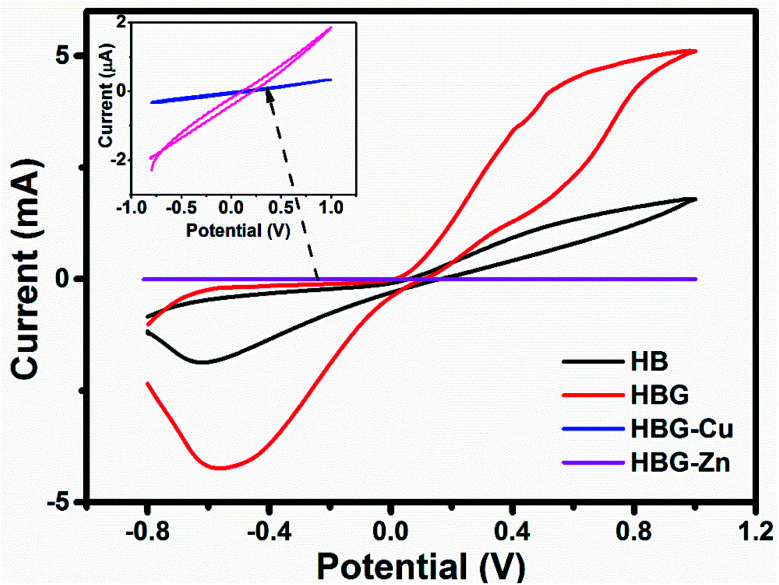
Cyclic voltammogram of polyacrylamide-based hydrogels at 40 mV s^−1^.

The cyclic voltammograms of polyacrylamide hydrogels used as working electrodes were carried out under applied redox potential ranging from −0.8 V to 1.0 V with a varying scan rate from 10 to 100 mV s^−1^. The cyclic voltammetry study of polyacrylamide-based hydrogels with 0.5 M H_2_SO_4_ as the electrolyte was carried out to determine the effect of reduced graphene oxide and transition metals on charge transfer activity. Fig. 3 shows a well-defined cyclic voltammogram hysteresis curve for HB and HBG, which is an indication of charge storage mechanism for HB and HBG. Due to the presence of electroactive materials Na^+^ and alumina silicates of clay, HB shows a peak at 0.8 V corresponding to oxidation, whereas a reduction peak at −0.68 V is due to the gain of electrons. After modification of HB with rGO, an anonymous increase in redox peak currents and shifting of redox peaks were found, indicating that the electrochemical properties of HBG increases. The increase is due to the conductive carbon network of rGO, which will promote effective ion transfer and electroactive area, and provide conductive bridge for electron transfer between the working electrode and the electrolyte.^35^ However, after further incorporation of transition metals during HBG synthesis, the redox properties abruptly decrease from 5.09 mA to 1.89 μA and 0.39 μA for HBG-Zn and HBG-Cu respectively, which indicates that the electrochemical properties of the fabricated hydrogels were decreased by transition metal loading into the hydrogel matrix. The increase in resistance and decrease in electrical conductivity are due to the complex formation between transition metals and the conductive framework of rGO. There is no peak current observed for HBG-Cu and HBG-Zn, *i.e.* but only weak current flow through the electrode material is observed. Such results indicate that the electron transfer through the transition metal-loaded polyacrylamide-based hydrogels is very slow. Transition metals dominated upon the redox properties of HBG. Moreover, the higher current capability or redox property of HBG-Zn as compared to HBG-Cu is due to high oxidation power of Zn compared to Cu. On this basis, we conclude that when the Cu and Zn metals are loaded into the hydrogel 3D structure during the synthesis, they reduce the electrochemical properties of HBG. Moreover, we infer from the results that a decrease in current intensity shows successful embedding during the synthesis of the working electrode. Capacitance calculated by the area under the curve is in such manner that HBG shows a greater capacitive behaviour than that of other polyacrylamide-based hydrogels.

An investigation regarding anodic and cathodic peak currents of the synthesized polyacrylamide-based hydrogels was performed. Fig. S4† shows anodic and cathodic peak variation with respect to the square root of the scan rate. The results indicate that there is an apparent increase in both anodic peak current (*I*_pa_) and cathodic peak current (*I*_pc_) with the increase in scan rate. Fig. S4† and Table 1 show slope values calculated for *I*_pa_ and *I*_pc_*vs.* square root of the scan rate. The slope value is in decreasing order as follows: HBG > HB > HBG-Cu > HBG-Zn. The larger the slope value, the greater will be the conductive property of the electrode material.

**Table 1:** Anodic and cathodic peak current of polyacrylamide-based hydrogels

Parameters		Polyacrylamide based hydrogels			
		HB	HBG	HBG-Cu	HBG-Zn
*I*_pa_	Slope	3 × 10^−4^	8 × 10^−4^	2 × 10^−7^	1 × 10^−7^
	Intercept	−0.0001	−0.0017	−8 × 10^−7^	1 × 10^−6^
	*R* ^2^	0.9772	0.8049	0.8781	0.9403
*I*_pc_	Slope	−4 × 10^−4^	−8 × 10^−4^	−2 × 10^−7^	−1 × 10^−7^
	Intercept	0.0005	0.0018	7 × 10^−7^	−1 × 10^−6^
	*R* ^2^	0.9854	0.8394	0.896	0.9668

Fig. S5† shows the peak-to-peak potential difference measured for all the fabricated polyacrylamide-based hydrogels at 100 mV s^−1^. It was investigated that the difference in anodic and cathodic peak potentials was in a descending order as follows: HBG < HB < HBG-Cu < HBG-Zn. This affirms that electric surface properties were greater in HBG, which in turn is greater than HB > HBG-Cu > HBG-Zn. It has been assumed that by incorporating transition metals, the electric properties of the synthesized hydrogels were decreased. HBG-Cu offered higher conductivity than that of HBG-Zn, which is due to the higher conductivity of Cu, *i.e.* 59.8 MS m^−1^, than that of Zn, *i.e.* 16.82 MS m^−1^.

In CV analysis, the total current measured under applied potential at different sweep rates is related to the sum of faradaic reaction on the exposed electrode area, diffusion controlled process and the current required to charge the double layer. An empirical description is expressed in eqn (1).

1 log *I* = log *a* + *b* log *γ*

where *I* is the anodic peak current and *γ* is the scan rate while both *a* and *b* are adjustable parameters. *b* is determined by the slope by plotting log *I vs.* log *γ* and is beneficial to provide information regarding the kinetic information of electrochemical reactions in polyacrylamide-based hydrogels. From the slope value expressed in Fig. S6,† it is inferred that the *b* value for HB, HBG, HBG-Cu and HBG-Zn is 0.564, 0.986, 0.891 and 0.225. HB and HBG-Zn values are closer to 0.5, which elucidates that the mechanism involved is a diffusion-controlled process. However, HB and HBG-Cu values are closer to unity, which illustrates that the process is adsorption controlled.^36^

#### 3.3.2 Impedance spectroscopy

**Fig. 4:**
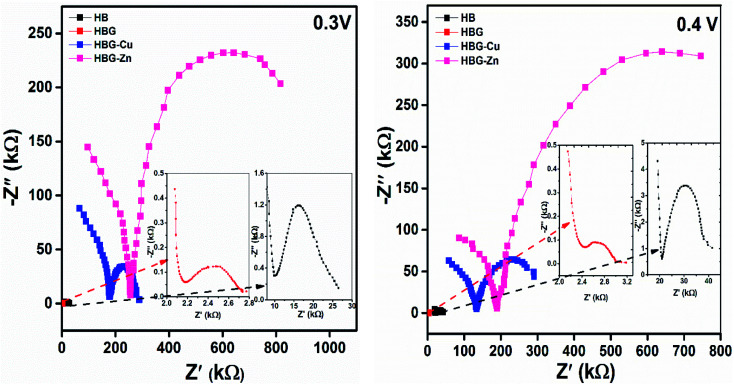
Nyquist plot of polyacrylamide-based hydrogels at 0.3 V and 0.4 V.

**Fig. 5:**
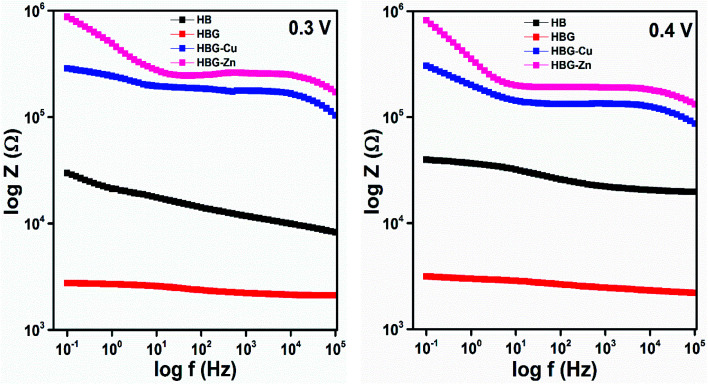
Bode plot (log *Z vs.* log *f*) of polyacrylamide-based hydrogels at 0.3 V and 0.4 V.

**Fig. 6:**
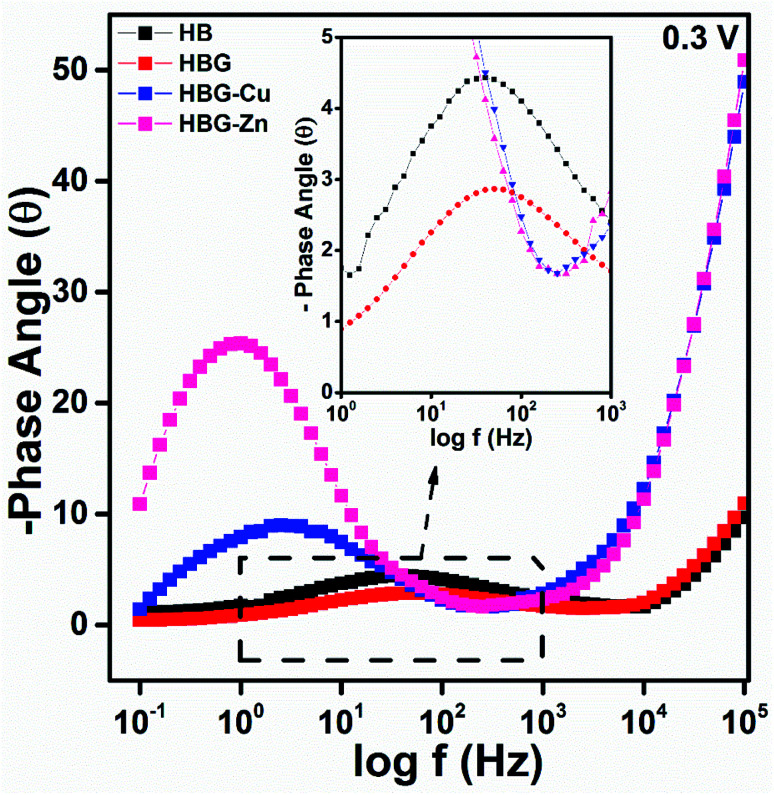
Bode plot (−phase angle *vs.* log *f*) of polyacrylamide-based hydrogels at 0.3 V.

In order to obtain in-depth knowledge about recombination and charge transport, impedance spectroscopic measurements were carried out. The Nyquist and Bode plots at different applied DC voltages *i.e.* 0.3 V and 0.4 V of polyacrylamide-based hydrogels are shown in Fig. 4–6. Spectroscopic measurements were performed at a frequency ranging from 0.1 Hz to 100 kHz at 0.01 AC Voltage. A high-frequency incomplete semicircle corresponds to the electrolyte reaction or solution resistance (*R*_s_). *R*_s_ is the resistance between the reference electrode and the working electrode, which was determined on real impedance at a higher frequency *f*_0_, whereas a complete semicircle at a low frequency deduces electrode reactions or electron transfer resistance (*R*_et_). *R*_et_ is equal to the diameter of the semicircle in the Nyquist plot. A semi-circle in the Nyquist plot indicated the electron transfer resistance, and has direct proportionality. From Fig. 4, no inclined slope was found at a low frequency, which means that there is no Warburg impedance.^37,38^ However, in the said study, there are three parameters present, namely, solution resistance, electron transfer resistance, and double layer capacitance. The capacitance was calculated from the Nyquist plot using eqn (2). Fig. S7† shows the best electrochemical circuit model fitted for the working electrodes, which clarifies that the circuit consists of solution resistance, electron transfer resistance, and double layer capacitance.2
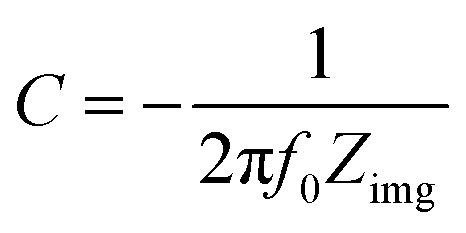


Using the Nyquist plot, at a higher frequency and 0.3 V, HB shows an *R*_s_ value (7935 Ω) larger than that of HBG (2077 Ω). The conducting framework of rGO in HBG will enhance the conducting nature and decrease the electrolyte resistance of HB. However, after further loading of transition metals, the *R*_s_ increases abruptly for HBG-Cu and HBG-Zn *i.e.* 65 466 Ω and 96 570 Ω, while decreasing the conductivity of the electrode material. Due to the blockage of active sites available for the transport of current, the ionic conductivity of the electrolyte (H_2_SO_4_) decreases. Zn and Cu in such mechanism act as insulators that block all the available electrochemical reaction sites. At a lower frequency, it is shown in Fig. 4 that electron transfer resistance between the working electrode and the electrolyte increases with the addition of transition metals. Ret is in such an order that HBG < HB < HBG-Cu < HBG-Zn, which is in correlation with the solution resistance, because due to the presence of Zn and Cu, high resistivity of transition metals will hamper the electron transfer between the working electrode and the electrolyte.

In a comparative study of polyacrylamide-based hydrogels with respect to measuring voltage *i.e.* 0.3 V and 0.4 V, it was observed that the real part of *Z* at a high frequency changes with respect to measuring voltage, which presumes that the working electrode (polyacrylamide based hydrogels) also shows resistance towards the charge transfer mechanism. HB and HBG shows an increase in *R*_s_, due to the fact that both the samples show a larger conductivity behaviour, whereas HBG-Cu and HBG-Zn show a decrease in *R*_s_ with respect to the increase in the measuring voltage. A smaller *R*_et_ suggests faster electron transfer, while a larger *R*_et_ suggests the least electron transfer. Fig. 4 shows the *R*_et_ for all transfer or higher charge transfer resistance at larger applied potentials, which will decline cell performance, indicating least feasibility for the accumulation of charges at the interface. Y. Xu *et al.*, reported the synthesis of electro-conductive hydrogels using PEG, PEDOT and PES substrates. The semi-circle obtained from the Nyquist plot has a larger diameter than that reported in the present work, which infers the most selectivity of HBG compared to the reported hydrogel.^39^

The capacitance calculated using eqn (2) for polyacrylamide-based hydrogels is expressed in Table 2. It has been depicted that the capacitance decreases with the increase in voltage for HB and HBG. While with the increase in potential, the capacitance increases with the rise in applied measuring voltage for HBG-Cu and HBG-Zn. The decrease and increase in capacitance are due to conductor and insulator behaviours of the fabricated hydrogels.

**Table 2:** Capacitance calculated from the Nyquist plot at different applied potentials

Applied voltage	HB	HBG	HBG-Cu	HBG-Zn
0.3 V	1.19 nF	3.64 nF	18.0 pF	10.9 pF
0.4 V	3.69 nF	3.35 nF	25.3 pF	17.5 pF

As depicted in Fig. 5, the impedance in HB is gradually decreased with the addition of rGO due to enhanced electron transfer mechanism of rGO as illustrated by the cyclic voltammetry and Nyquist plot. However, after further addition of transition metals, impedance abruptly increases, enhancing the resistance towards the electron transfer. However, the higher impedance of HBG-Zn than that of HBG-Cu is due to the fact that the standard electrical resistivity at 293 K of Zn is 59.6 nΩm, whereas for Cu it is 16.94 nΩm. As Cu has least resistivity as compared to Zn, the impedance follows the order of HBG-Cu < HBG-Zn. Impedance relating to frequency shows that all the hydrogels show resistance at high frequencies while capacitance at lower frequencies. Table 3 shows the impedance at lower and higher frequency ranges at 0.3 V and 0.4 V. It has been depicted that for both voltages, the impedance at lower and higher frequencies is in descending order as follows: HBG-Zn > HBG-Cu > HB > HBG. The impedance calculated by Y. Xu *et al.*, for electro-conductive hydrogels is in the range of 10^4^, whereas in our analysis for HBG, the impedance is 2751 and 2115 Ω at high and low frequencies, which is very low than that of the reported material.^39^

**Table 3:** Impedance from the Bode plot at different applied potentials

Frequency	HB		HBG		HBG-Cu		HBG-Zn	
	0.3 V	0.4 V	0.3 V	0.4 V	0.3 V	0.4 V	0.3 V	0.4 V
100 kHz	8289 Ω	19 775 Ω	2115 Ω	2198 Ω	103 424 Ω	85 551 Ω	172 498 Ω	132 587 Ω
0.1 Hz	29 911 Ω	39 648 Ω	2751 Ω	3142 Ω	299 038 Ω	296 223 Ω	878 527 Ω	819 350 Ω

For HB and HBG, the impedance increases, while for HBG-Cu and HBG-Zn, the impedance decreases with the increase in applied measuring voltage. The increase in impedance and *R*_s_ is according to eqn (3), *i.e.* the applied voltage is directly proportional to the impedance. Therefore, in such aspects, an increase in the applied measuring voltage increases the impedance. The decrease in impedance with the increase in potential is due to the fact that Cu and Zn act as insulators enhancing the resistance for the transfer of electrons. Therefore, when the applied potential is increased, the barrier breakdown occurred, and thus, *R*_s_ and impedance decrease with the increase in potential.

3 *V* = *IZ*

The impedance phase curves for all polyacrylamide-based hydrogels through the entire range from 0.1 Hz to 100 kHz are displayed in Fig. 6. The peak and width of the phase angle can be used for determining the resistance property of the material. Electrode material having low phase angle peak at the high-frequency range, least will be the resistance. Whereas an electrode material has high phase angle peak at low frequency, the maximum will be the resistance. HB and HBG show approximately the same value at all frequencies, while HBG-Cu and HBG-Zn show a similar pattern in the whole frequency range. Moreover, the phase angle for HBG-Zn moves slightly towards a low frequency as compared to HBG-Cu. As there is a great discrimination between the fabricated hydrogels, the phase angle will be used for estimating the super capacitive and resistive behaviours. The phase angle peak observed for HB is 4.44°, whereas for HBG, the peak is 2.88°. However for HBG-Cu and HBG-Zn, the peak is 9.04° and 25.68°. From the plot of −phase angle *vs.* log *f*, we also deduce that besides the phase angle peak, there is a shift in frequency for all the fabricated hydrogels. HB, HBG, HBG-Cu and HBG-Zn show a phase angle peak at 39.21 Hz, 51.16 Hz, 2.65 Hz and 0.813 Hz. It was inferred that HBG shows a higher capacitive behaviour due to a least phase shift value at a higher frequency compared to other fabricated hydrogels. The order of shifting at a lower frequency for HBG-Cu and HBG-Zn also indicates the increase in resistivity. Apparently, this indicates that a greater roughness was found in the conduction of fabricated hydrogels containing transition metals.

#### 3.3.3 Galvanostatic charge–discharge

Galvanostatic charge–discharge curves of the synthesized hydrogels were determined to elucidate their energy storage performance. The GCD curves were recorded in a three-electrode cell using the hydrogel as the working electrode, gold as the counter electrode and Ag/AgCl as the reference electrode. To investigate the effect of current upon charging and discharging, different charge/discharge curves were recorded at a specified current densities, namely, 0.5 A g^−1^, 1.0 A g^−1^ and 1.5 A g^−1^. From the experimental data, specific capacitance, energy density and power density of the fabricated hydrogel were calculated using eqn (4)–(6):4
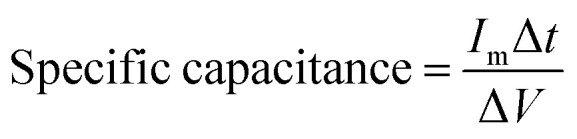
5
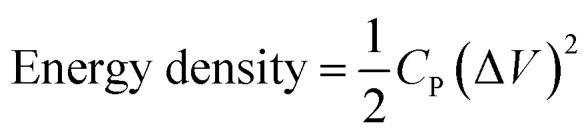
6
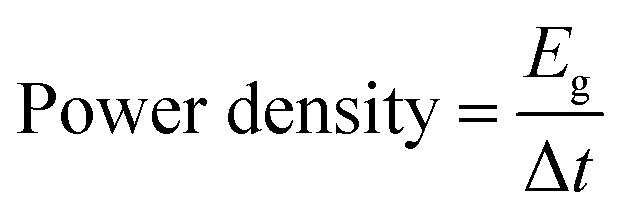
where *I*_m_ is the given current in charging/discharging, Δ*t* is the change in time in the discharging mode while Δ*V* is the change in potential.

**Fig. 7:**
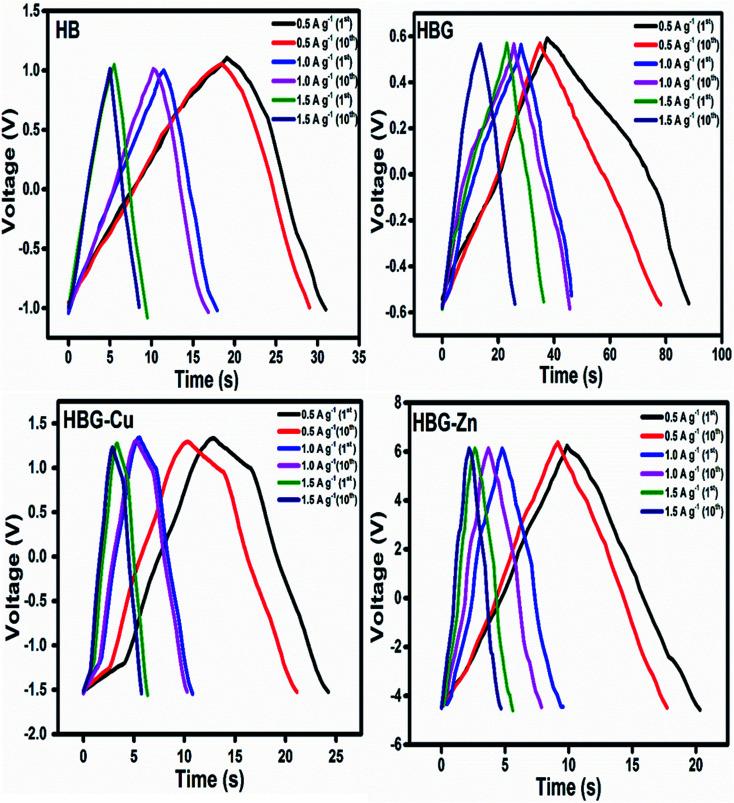
Galvanostatic charge–discharge curves of the fabricated hydrogels at different current densities for 1st and 10th cycles.

The charge discharge property of all fabricated gels was determined, which is illustrated in Fig. 7. All hydrogels show a difference in their charging and discharging voltages that depend on the conductance and resistance of the working electrode as already illustrated in CV and EIS analysis. The HBG charge has a very low voltage due to its low resistance; however, other fabricated gels show an increase in their charging voltage due to maximum resistance offered to the flow of electrons or ions.

The discharge time calculated by GCD shows that HBG has a longer discharge time than that of other fabricated gels, *i.e.* without rGO and rGO with transition metals, indicating a higher charge storage than that of other gels. Due to the long discharge time, HBG shows a larger specific capacitance, *i.e.* 22.923 F g^−1^, which means that rGO caused an increase in *C*_P_ as compared to other gels that have specific capacitances of 3.25 F g^−1^, 2.02 F g^−1^, and 0.49 F g^−1^ for HB, HBG-Cu and HBG-Zn respectively. The results obtained show similar behaviors to those achieved by CV and EIS that indicate a rapid current response with excellent capacitance. *C*_P_ of the fabricated gels was also calculated as a function of different applied current densities. The results indicate that with the increase in current density, Δ*t* decreases, which is due to high discharge current from the electrode material; as a result, less time is required for a gel to be discharged, resulting in a decrease in capacitance or stored charge. At different applied current densities, all fabricated gels show a decrease in their capacitance due to the fast discharge of the gel. Moreover, the number of cycles was investigated at different current densities. The result indicates that with the increase in the number of cycles, the peak shift decreases in Δ*t* occurred due to the decreasing efficiency of the electrode material.

**Fig. 8:**
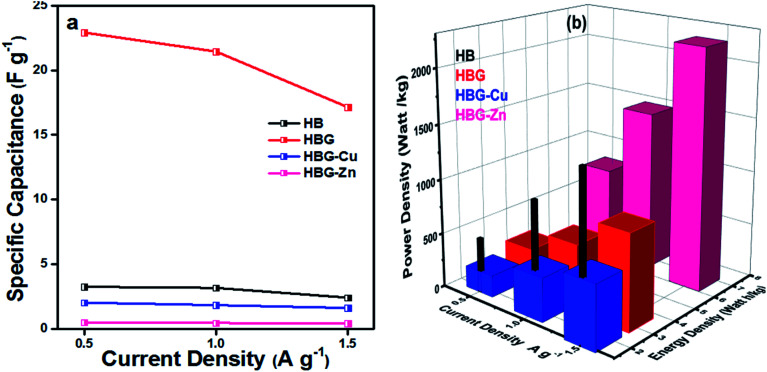
Effect of current density on specific capacitance (a) and energy density and power density (b) of polyacrylamide-based hydrogels.

Fig. 8a shows specific capacitance calculated using eqn (4) at different discharge currents. The result indicates that HBG shows a decrease in specific capacitance from 22.92 to 17.14 F g^−1^ and retains 75% of its capacitance as compared to other fabricated gels, as depicted in Fig. 8a. Similarly, with the increase in the number of cycles up to 10, HBG retains a *C*_P_ of 83% as compared to other cycles having 80%, indicating the high stability of HBG as compared to other polyacrylamide-based hydrogels. Table 4 shows some of the available working electrodes and their specific capacitance, which concludes that the synthesized HBG has a high energy storage capability as compared to the previously reported electrode materials.

**Table 4:** Comparison of electrochemical properties of hydrogel-based electrodes

Author	Hydrogel	Current density	Specific capacitance	Ref.
C. Liu *et al.*	60% poly (NIPAM-GO-AA)	0.5 A g^−1^	12 F g^−1^	40
J. Kalupson *et al.*	Carbon nanotube and hydrogel membrane	0–50 A g^−1^	11 F g^−1^	41
Juan Du *et al.*	ANI/PVA/TiO_2_ hybrid hydrogel	1 A g^−1^	14 F g^−1^	42
Chien-Chung Shih et. Al.	PEDOT:PSS/PVA/PMAA	0.5 A g^−1^	7.57 F g^−1^	43
Faizan *et al.*	Polyacrylamide	0.5 A g^−1^	22.923 F g^−1^	Current study

Fig. 8b shows the energy density and power density of fabricated gels at different current densities. The energy density and power density calculated from eqn (5) and (6) presume that *E*_g_ decreases with the increase in current density, while *P*_g_ increases with the increase in applied current density. Hydrogels with a maximum capacitance will have maximum *E*_g_ and *P*_g_. However, in the present study, *E*_g_ and *P*_g_ noticed for HBG-Zn are maximum as compared to other fabricated hydrogels. The peculiar behavior of HBG-Zn is due to the porous surface, which enhances the ion mobility across the polymer matrix when high voltage is applied across the circuit, *i.e.* up to 6 V, as illustrated in Fig. 7. Although the ion mobility is maximum and, hence, energy density is high, due to the insulating nature of HBG-Zn, it will release all its stored energy instantly. However, if we look at HBG, the values of *C*_P_, *E*_g_ and *P*_g_ are maximum, making it selective to be used for electrode materials. HB and HBG show both electrochemical double layer capacitance and pseudocapacitance because HB and HBG show redox properties as well as energy and power density values in the EDLC region. However, HBG-Cu and HBG-Zn show EDLC behavior because they do not show redox properties, but the charges accumulated on the working electrode by the generation of a double layer.

**Fig. 9:**
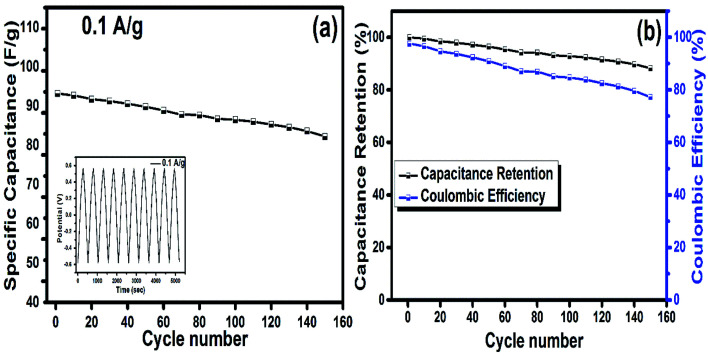
Specific capacitance *vs.* number of cycles (a), and cyclic stability and coulombic efficiency of HBG at 0.1 A g^−1^ (b).

The cyclic stability of the electrode is a significant indication for the flexible electrode to be used in real applications. Therefore, the hydrogel loaded on MBA and rGO was tested for 150 cycles in a potential range at a current density of 0.1 A g^−1^. The results from Fig. 9a indicated that the specific capacitance calculated was 93.16 F g^−1^, while with continuous charging and discharging of the flexible electrode, its charge storage capability decreases and, thus, attains a *C*_P_ of 82.18 F g^−1^ after 150 cycles. Fig. 9b infers the high stability of the HBG flexible electrode, having a capacitance retention up to 88.28% and a coulombic efficiency up to 77.2% after 150 cycles.

#### 3.3.4 Conductivity measurement

**Fig. 10:**
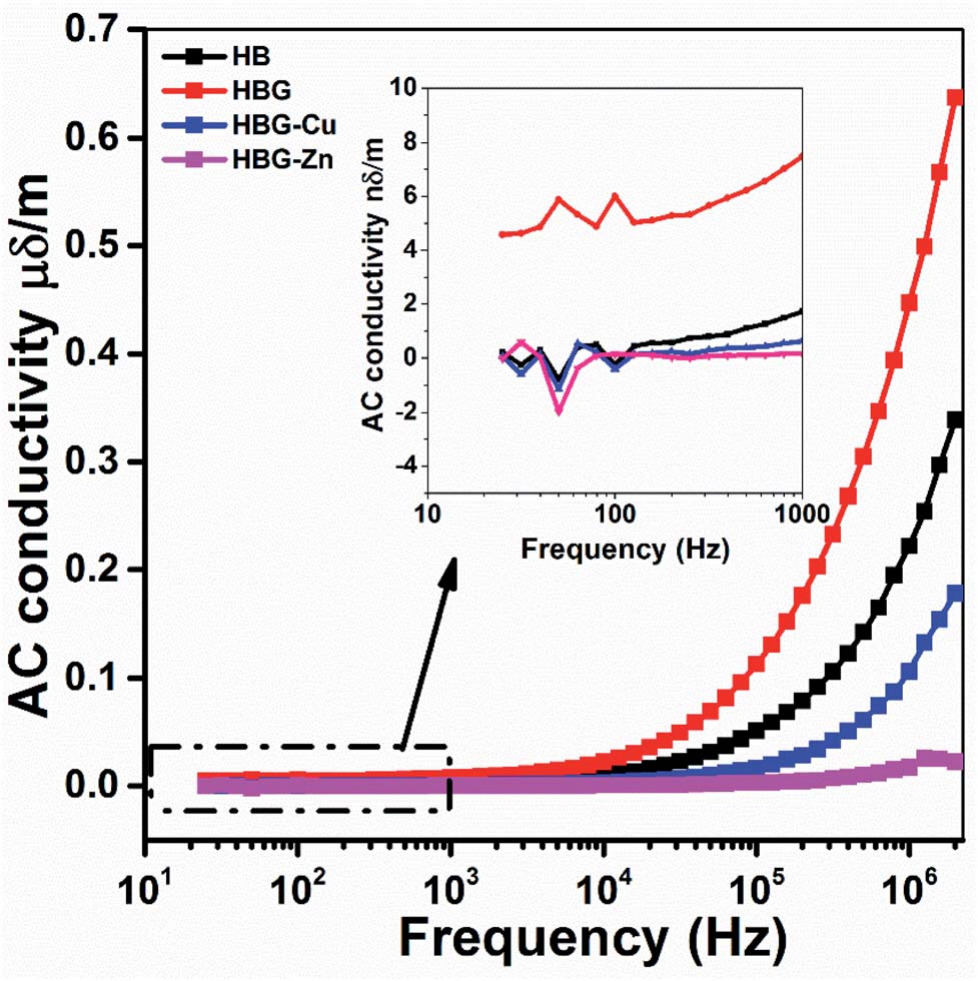
Variation in AC conductivity as a function of frequency for polyacrylamide-based hydrogels.

The electrical conductivity of the fabricated hydrogels was measured at 30 °C. Fig. 10 shows the variation in AC conductivity as a function of applied frequency on polyacrylamide hydrogels. The results indicate that at low frequency, the AC conductivity is almost the same, *i.e.* 0.5 nδ m^−1^, which increases with the increase in frequency. At a high frequency range, the ac conductivity of HBG is 0.67 μδ m^−1^, which is larger than that of other fabricated gels, *i.e.* 0.34, 0.17, and 0.0022 μδ m^−1^ for HB, HBG-Cu, and HBG-Zn respectively. The increase in conductivity with frequency is due to the polymeric semiconductor response. The AC conductivity of HBG as compared to HB is due to the introduction of a conducting carbon framework of reduced graphene oxide. However, after further *in situ* incorporation of transition metals, the agglomeration of transition metals (Cu and Zn) with graphene oxide blocks all the available sites responsible for current flow, which results in a decrease in conductivity at high frequencies. As the conductivity analysis was carried out using a Keithley LCR meter, polymer hydrogels were dried completely and then ground to prepare pellets for analysis. In such aspect, due to the removal of water and rough texture, the conductivity is extremely low.

#### 3.3.5 Hydrogel response towards external applied current

Fig. S8† shows the light response towards the applied DC current. It has been shown that when the circuit was connected with a steel terminal, the light intensity was much higher, but when hydrogels were placed in between the terminals, the light intensity drops. The drop is due to the fact that steel is a higher conductor of electric current, but has tough nature, which was resolved by the use of hydrogels. Although the light intensity was lower in case of HB, it was enhanced in a HBG-connected circuit. Moreover, the intensity can be further improved by increasing the concentration of GO in the polyacrylamide-based hydrogels for further studies. However, in case of HBG-Cu and HBG-Zn, the light intensity decreases as compared to that of HBG due to the resistive nature of hydrogels.

### 3.4 Mechanical analysis

#### 3.4.1 Oscillatory frequency sweep

Oscillatory measurements were carried out in order to infer about the visco-elastic property of the synthesized hydrogel. Visco-elastic moduli represent storage moduli (*G*′) that infer the amount of energy stored, whereas loss moduli (*G*′′) characterize the energy dissipated by the flow behavior.

**Fig. 11:**
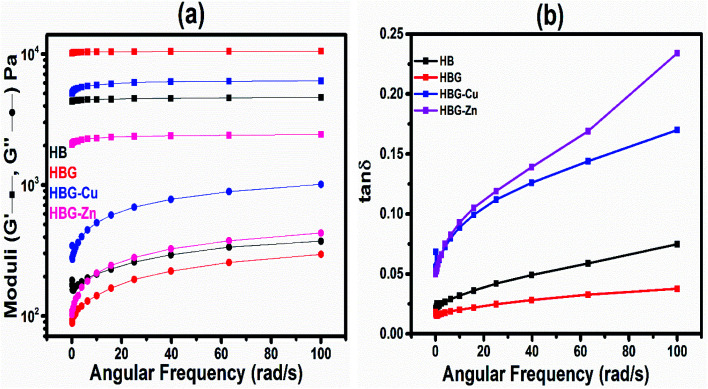
Moduli (a) and tan *δ* (b) as a function of angular frequency.

Fig. 11a shows the plot of storage and loss moduli as a function of angular frequency in the range of 0.1–100 s^−1^. The results indicated that in the whole frequency range, storage moduli are greater than loss moduli. *G*′ > *G*′′ infers the more elastic nature of fabricated hydrogels than the viscous nature. Furthermore, comparison among the synthesized hydrogels reveals that HB shows a storage modulus of 4.66 kPa at a high frequency, which is enhanced to 10.52 kPa by the addition of a physical cross-linker, *i.e.* graphene oxide, indicating the more elastic nature. The enhancement in *G*′ and *G*′′ is thought to be dual cross-linking, *i.e.* chemical and physical mechanisms by MBA and GO. However, by *in situ* incorporation of transition metals, *i.e.* Cu^2+^ and Zn^2+^, the storage moduli dropped to 6.22 kPa and 2.43 kPa for HBG-Cu and HBG-Zn. The drop in storage moduli is attributed to the complexation of graphene oxide with transition metals, which can also be observed by the agglomeration of graphene oxide particles when Cu^2+^ and Zn^2+^ were loaded during synthesis. Thus, the cross-linking mechanism was altered, which was enhanced by graphene oxide. The results indicated the more suitability of HBG for flexible electrode application because of high storage and least loss moduli compared to other fabricated hydrogels, as mentioned in Table 5.

**Table 5:** Comparison of the mechanical properties of various polyacrylamide-based hydrogels

Author	Hydrogel	Cross-linker	Storage moduli	Ref.
Bakhtawara *et al.*	Poly (AAm-co-AAc-co-AMPS)	MBA	3.2 kPa	33
Faizan *et al.*	SA-poly (MAA-co-AMPS)	Borate anhydrous	2.9 kPa	44
Ali. I. *et al.*	Poly ethylene glycol modified-polyacrylamide	MBA	0.1 kPa	45
Shah. L. A. *et al.*	Poly(*N*-vinyl formamide-co-acrylamide)	MBA	0.2 kPa	46
Cheng. W. M. *et al.*	Poly(acrylic acid-co-acrylamide)	MBA	1.8 kPa	47
Faizan *et al.*	Polyacrylamide	MBA & rGO	10.52 kPa	Current study

The damping factor is the characteristic property of visco-elastic materials. Fig. 11b shows that in the whole frequency range, the value of tan *δ* is less than 1, which infers the more elastic nature than the viscous nature. Moreover, the closer the value to 0, the maximum will be the elasticity. The results indicate that the tan *δ* value for HBG is closer to zero, implying its high mechanical strength compared to other synthesized hydrogels.

#### 3.4.2 Influence of applied stress on mechanical strength

Video 1† shows a lab-based experiment in which stress has been applied on the cross-sectional area in order to determine whether the fracture point appears at ordinary stress while using wearable electronics as flexible electrode materials. The result indicated that HBG shows no fracture point when stress is applied on it, which makes it a suitable candidate to be used in portable and robotics technology.

## 4. Conclusion

A series of electrochemical studies have been carried out to elucidate the effect of reduced graphene oxide and transition metals, namely, Cu^+2^ and Zn^+2^ on polyacrylamide-based hydrogels synthesized *via* free radical polymerization using ammonium pershuplate as an initiator. The electrochemical studies revealed a higher impact of current and voltage on the electrochemical properties of the fabricated hydrogels. The hydrogel composed of sodium borate anhydrous and reduced graphene oxide shows higher electrochemical behaviors having a peak potential difference, *i.e.* 1.48438 V, affirming maximum electrical surface properties of the fabricated hydrogel, and will be used on an industrial scale for energy storage devices and flexible electrodes. HBG shows least solution resistance and phase shift value, *i.e.* 2100 Ω and 2.88°, which are quite lesser than those of other fabricated hydrogels, which show phase shift values of 4.44°, 9.04° and 25.68° for HB, HBG-Cu and HBG-Zn. The specific capacitance calculated for HBG is 22.923 F g^−1^ and its capacitance is retained up to 83.16% after 10 cycles, whereas the AC conductivity of HBG is higher than that of other fabricated hydrogels, *i.e.* 0.67 μδ m^−1^. HBG shows a maximum storage modulus of 10.52 kPa and a tan *δ* value lower than 1, indicating the more elastic nature of the synthesized polymer. The hydrogel specific capacitance can be further enhanced using transition metals *via* an *ex situ* mechanism. The fabricated hydrogels were flexible in nature having high ion mobility.

## Conflicts of interest

No conflicts of interest exist to declare.

## Supplementary Material

RA-012-D2RA02391A-s001

RA-012-D2RA02391A-s002
