# Supplementary material for: Effects of Cu2+/Zn2+ on the electrochemical performance of polyacrylamide hydrogels as advanced flexible electrode materials
Source: RSC Adv. 2022 Jun 30;12(30):19072–85. doi: 10.1039/d2ra02391a (PMC9244644; doi:10.1039/d2ra02391a)
Supplement: RA-012-D2RA02391A-s002 [file RA-012-D2RA02391A-s002.pdf]

Electronic Supplementary Material (ESI) for RSC Advances.
This journal is © The Royal Society of Chemistry 2022

Effect of Cu^{2+}/Zn^{2+} on electrochemical performance of polyacrylamide hydrogel as advanced flexible electrode materials

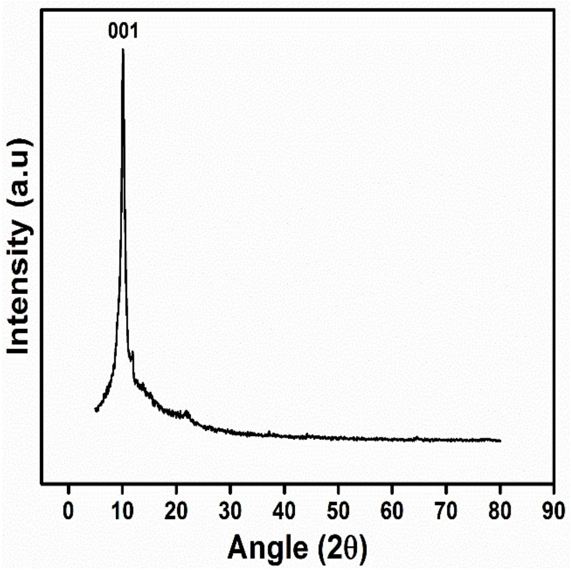

Fig. S1: XRD pattern of Graphene oxide

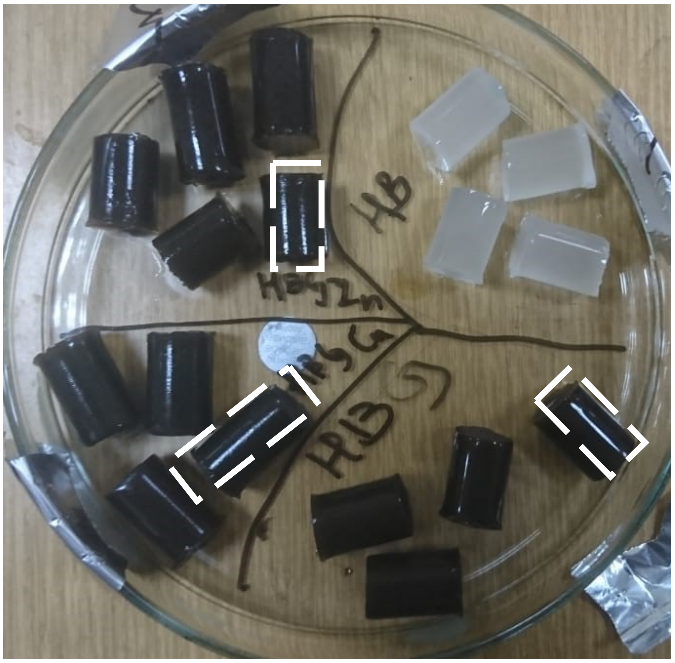

Fig. S2 Light reflection from surface of Polyacrylamide based Hydrogels before reduction

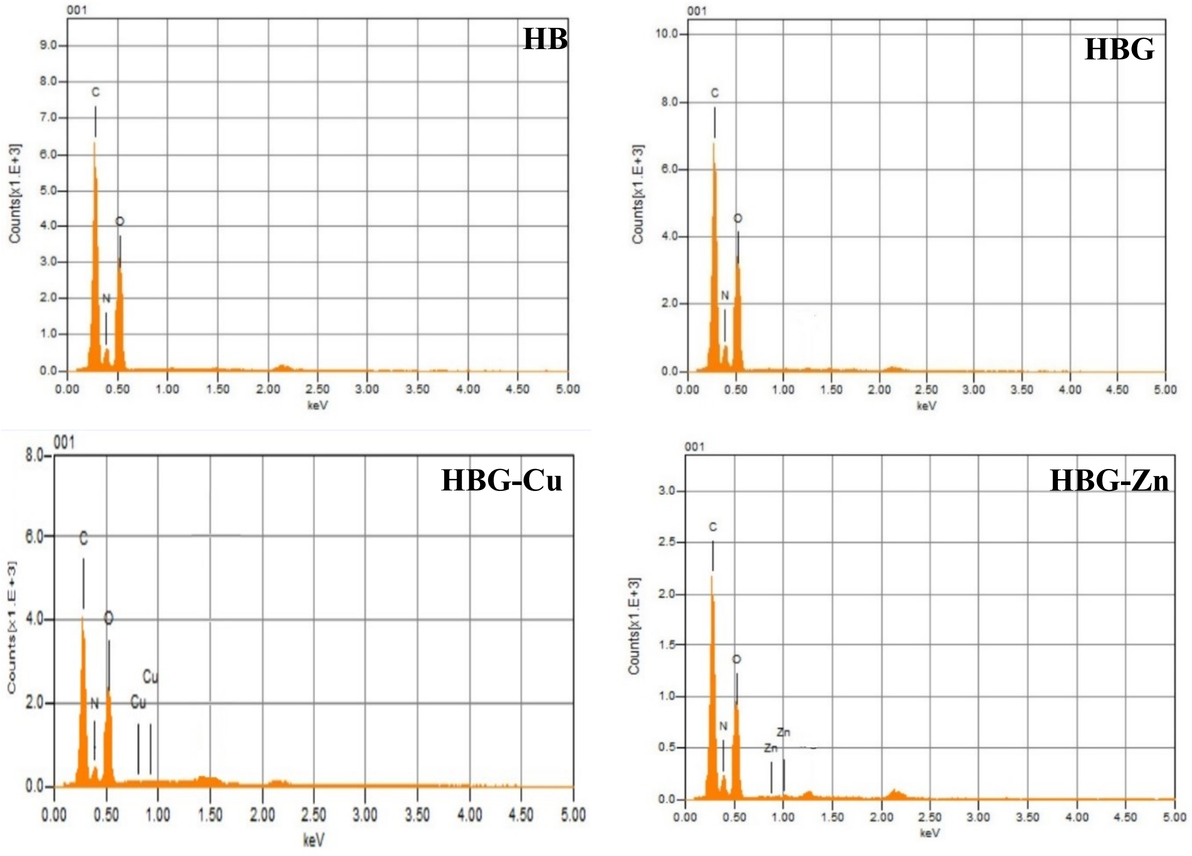

Fig. S3 EDX spectra of multi cross-linked polyacrylamide hydrogels

Table S1 Atomic percentage of each atom measured through EDX analysis for prepared multi cross-linked polyacrylamide hydrogels

**Hydrogels**	**Atomic %**				
	**C**	**N**	**O**	**Cu**	**Zn**
**HB**	61.26	6.21	32.53	-	-
**HBG**	61.10	6.92	31.98	-	-
**HBG-Cu**	63.23	6.30	30.43	0.04	-
**HBG-Zn**	63.48	6.27	30.21	-	0.04

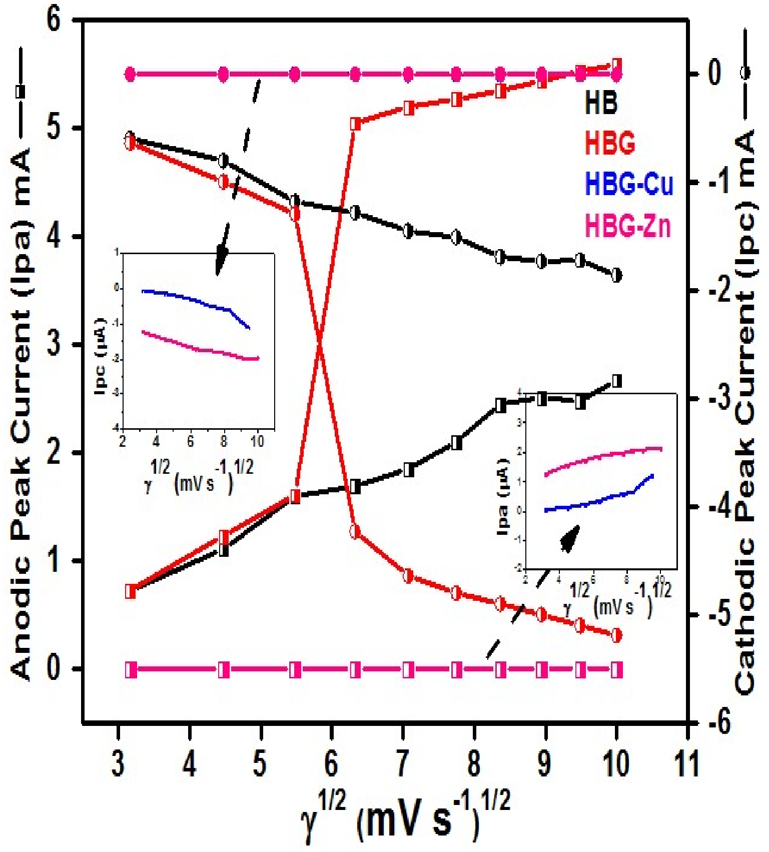

Fig. S4 Anodic and Cathodic peak current of polyacrylamide based hydrogels

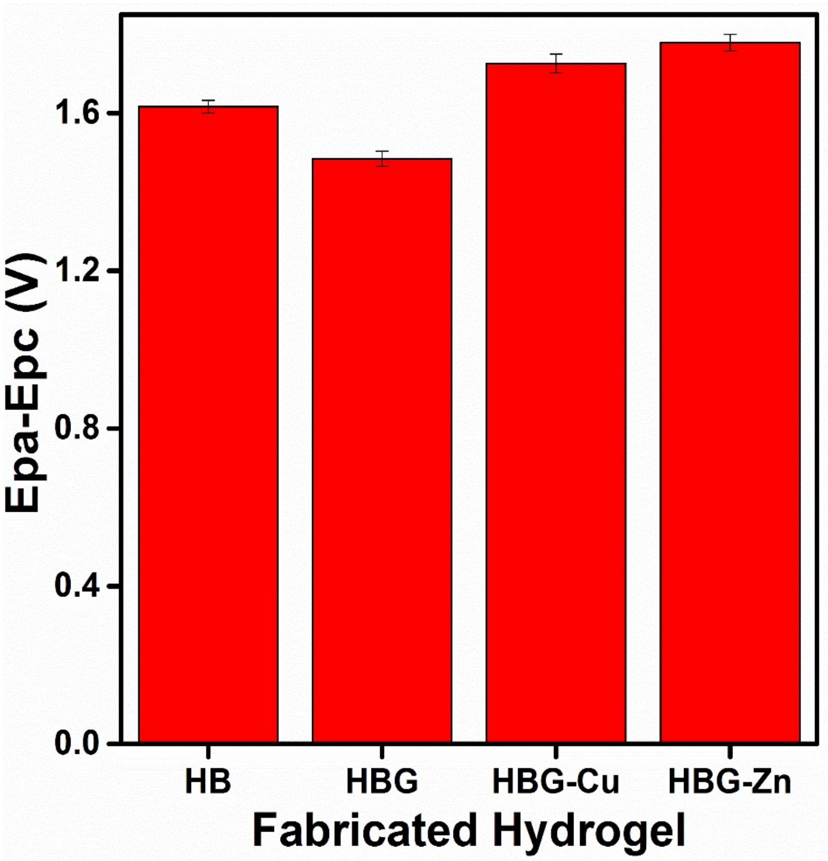

Fig. S5 Peak potential separation of polyacrylamide based hydrogels

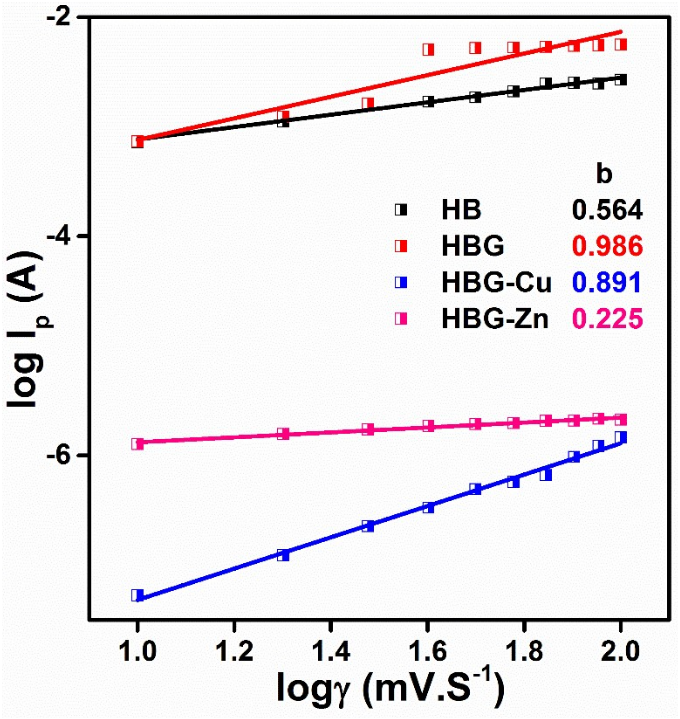

Fig. S6 Peak current at different sweep rates

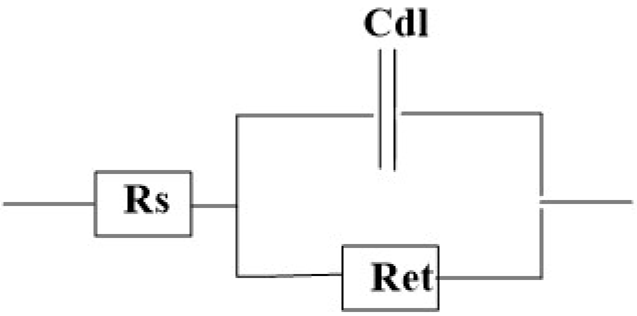

Fig. S7 Electrochemical circuit fit model for Polyacrylamide based hydrogels

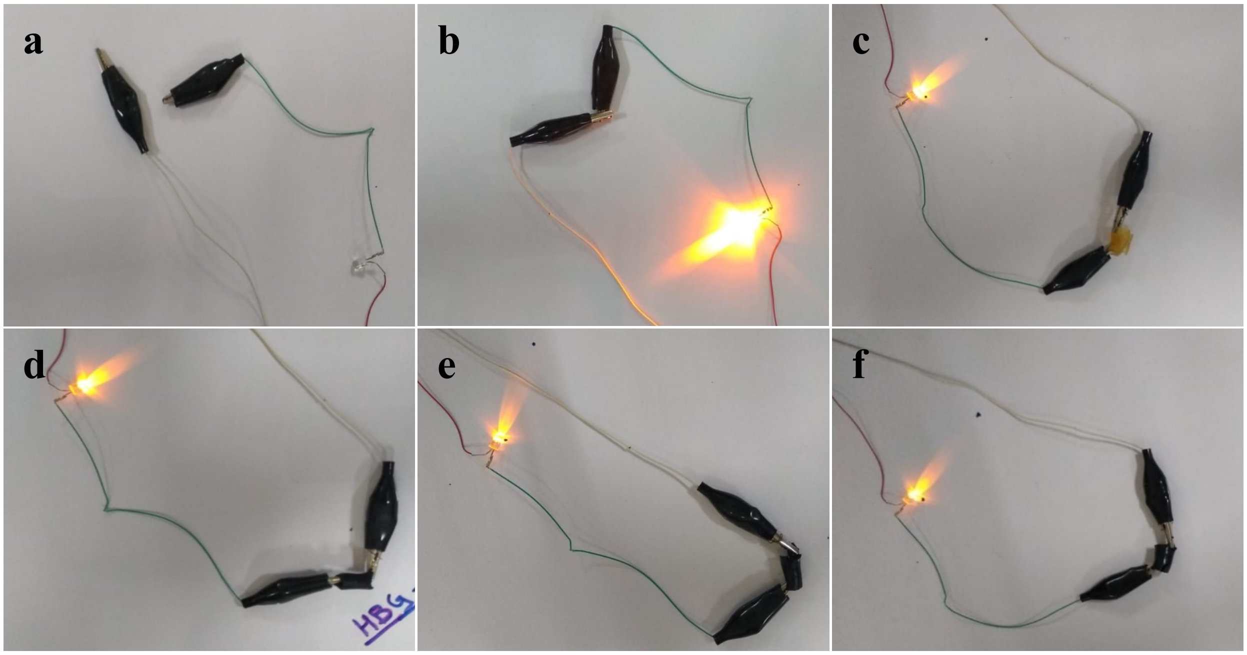

Fig. S8 Light response towards applied external DC current i.e. not connected (a), terminals connected in the absence of Hydrogel (b), and in the presence of Hydrogels i.e. HB (c), HBG (d), HBG-Cu (e) and HBG-Zn (f)
